# Status of Compassionate, Respectful, and Caring Health Service Delivery: Scoping Review

**DOI:** 10.2196/30804

**Published:** 2022-02-07

**Authors:** Adane Nigusie, Berhanu F Endehabtu, Dessie Abebaw Angaw, Alemayehu Teklu, Zeleke Abebaw Mekonnen, Marta Feletto, Abraham Assan, Assegid Samuel, Kabir Sheikh, Binyam Tilahun

**Affiliations:** 1 Department of Health Education and Behavioural Sciences Institute of Public Health, College of Medicine and Health Sciences University of Gondar Gondar Ethiopia; 2 Department of Health Informatics Institute of Public Health, College of Medicine and Health Sciences University of Gondar Gondar Ethiopia; 3 Department of Epidemiology and Biostatics Institute of Public Health, College of Medicine and Health Science University of Gondar Gondar Ethiopia; 4 Department of Pediatrics and Child Health School of Medicine, College of Medicine and Health Science University of Gondar Gondar Ethiopia; 5 Alliance for Health Policy and Systems Research World Health Organization Geneva Switzerland; 6 Human Resources Administration Directorate Ministry of Health Addis Ababa Ethiopia

**Keywords:** compassionate, respectful, caring, CRC, health care delivery

## Abstract

**Background:**

A compassionate, respectful, and caring (CRC) health professional is very important for human-centered care, serving clients ethically and with respect, adhering to the professional oath, and serving as a model for young professionals. As countries try to achieve universal health coverage (UHC), quality delivery of health services is crucial. CRC health care is an initiative around the need to provide quality care services to clients and patients. However, there is an evidence gap on the status of CRC health care service delivery.

**Objective:**

This scoping review aimed to map global evidence on the status of CRC health service delivery practice.

**Methods:**

An exhaustive literature review and Delphi technique were used to answer the 2 research questions: “What is the current status of CRC health care practices among health workers?” and “Is it possible for health professionals, health managers, administrators, and policy makers to incorporate it into their activity while designing strategies that could improve the humanistic and holistic approach to health care provision?” The studies were searched from the year 2014 to September 2020 using electronic databases such as MEDLINE (PubMed), Cochrane Library, Web of Science, Hinari, and the World Health Organization (WHO) library. Additionally, grey literature such as Google, Google Scholar, and WorldWideScience were scrutinized. Studies that applied any study design and data collection and analysis methods related to CRC care were included. Two authors extracted the data and compared the results. Discrepancies were resolved by discussion, or the third reviewer made the decision. Findings from the existing literature were presented using thematic analysis.

**Results:**

A total of 1193 potentially relevant studies were generated from the initial search, and 20 studies were included in the final review. From this review, we identified 5 thematic areas: the status of CRC implementation, facilitators for CRC health care service delivery, barriers to CRC health care delivery, disrespectful and abusive care encountered by patients, and perspectives on CRC. The findings of this review indicated that improving the mechanisms for monitoring health facilities, improving accountability, and becoming aware of the consequences of maltreatment within facilities are critical steps to improving health care delivery practices.

**Conclusions:**

This scoping review identified that there is limited CRC service provision. Lack of training, patient flow volume, and bed shortages were found to be the main contributors of CRC health care delivery. Therefore, the health care system should consider the components of CRC in health care delivery during in-service training, pre-service training, monitoring and evaluation, community engagement, workload division, and performance appraisal.

## Introduction

### Background

Compassionate, respectful, and caring (CRC) health care delivery is an essential element for health workforces that builds a positive environment and intimacy among health care professionals, patients, and families. Worldwide, improving the quality of care using the limited skilled health workforce is a major challenge facing the health care system [1]. Universal health coverage (UHC) is realized when everyone has access to quality essential health care services with financial risk protection [2,3]. However, each year, almost half of the world’s population cannot access the needed health services, and millions of people are forced into extreme poverty due to catastrophic out-of-pocket health expenditures [4].

An effective health system helps to promote UHC through the provision of equitable, quality, responsive, efficient, and resilient health services [5-7]. The health workforce is one of the 6 building blocks that make a health system function [8].

CRC health professionals are crucial to the strategy designed to improve the quality of care and which has several benefits for both the provider and end service users. Supporting a movement toward creating a CRC health workforce is in the agenda of most African countries [9-11]. In order to realize this vision, intensive effort by leaders and health managers at all levels of the health sector [12,13] as well as supporting systems and structures [14] are required.

Compassion and acting to relieve concerns, pain, distress, and suffering are fundamental to health care; these define the higher purpose of the health care system. Respect for people goes beyond accepting the notion or attitude that people have autonomous choice; rather, it is treating others in such a way that enables them to make the choice. Respecting the patient’s right to self-determination, that is supporting decisions that reflect the patient’s personal beliefs, values, interests, and problems, is thus central [12,15].

Even though a lot has been done globally to improve the health status of the population, it is still failing at a fundamental level [16-18]. The qualities of caring, respect, and compassion, which form the basis of care delivery and the human aspects that define it, have been replaced by a primary focus on pathways, tasks, and documentation [12,19].

Hence, this review aimed to assess CRC practices in health care delivery and highlight the reality of the situation. The goal was to identify key issues in developing guidance for health professionals, health managers, administrators, and policy makers, to inform and refine strategies that could improve the humanistic and holistic approach of health care provision.

### Rationale for the Review

Ensuring CRC health services is one of the most important facilitating factors to increase access to and the continuum of care. However, challenges to implementing CRC health services persist and reflect large disparities across geographic areas and population groups [20-22]. Little is known about the status of CRC practices in the health systems and their underlying determinants.

Hence, this scoping review of the literature aimed to draw out the evidence regarding the current status of CRC health service delivery and design a CRC implementation strategy. In addition, this review of the literature aimed to identify the possible health care practices research areas that need critical insight and further investigation.

## Methods

### Literature Search and Search Methods

Our review aimed to identify the available evidence to provide an overview of the scoping review objectives of the current state of knowledge on CRC health service practices. This review was conducted following the PRISMA-ScR (Preferred Reporting Items for Systematic reviews and Meta-Analyses extension for Scoping Reviews) checklist, and it was guided by Joanna Briggs Institute (JBI) scoping review guidance [23,24]. A search was conducted for published and unpublished (grey) literature on the research area in the following electronic databases: MEDLINE (PubMed), Cochrane Library, Web of Science, Hinari, and the World Health Organization (WHO) library. Moreover, grey literature was searched on Google, Google Scholar, and WorldWideScience. All studies published from 2014 to September 2020 were considered. We used different combinations of keywords and text to build the search strategy and identify relevant articles. The searching techniques for PUBMED considered Boolean operators with the following search terms: “((compassion*) OR compassionate OR concern) OR empathy) OR kindness) OR consideration)) AND ((Respect*) OR Respectful) OR deferent*) OR reverent*) OR polite*) OR courteous) OR considerate) OR civil)) AND ((caring) OR care OR) kind) OR thoughtful) OR gentle) OR helpful) OR considerate) OR compassionate) OR concerned) OR loving)) AND ((health professional* OR health personnel* OR health care provider* OR health care worker* OR nurses OR Midwives* OR pharmacists OR physicians OR health care worker*)) AND ((behaviour) OR performance) OR actions) OR deeds) OR activities) OR manners) OR conduct)) AND ((health care delivery OR delivery of health care OR performance OR behaviour OR health care system OR health care systems))”.

To identify the potentially relevant literature, a hand search was also conducted of the references of the included studies and websites such as the WHO and Directory of Research on CRC. Potentially relevant grey literature was identified through targeted searches of dissertations, theses, and conference abstracts (EMBASE Conference Abstracts, Conference Proceedings Citation Index—Science and Social Science & Humanities).

### Scoping Review Research Question

We used a population, concept, and context (PCC) framework developed by the JBI to determine the eligibility of our primary research question. The primary research question for this scoping review was: “What is the current status of CRC health care practices among health workers?” The other research question was “Is it possible for health professionals, health managers, administrators, and policy makers to incorporate it into their activity while designing strategies that could improve the humanistic and holistic approach to health care provision?” This study used the PCC format (Table 1) to align the study selection with the aforementioned research question.

**Table 1:** Eligibility of studies according to the participant, concept, and context (PCC) framework.

Criteria	Elements
P: participants	All categories of health professionals involved in health service delivery in all levels of public, private, or other sectors of health care
C: concept	Studies that explored compassionate, respectful, and caring behaviors by health professionals in different forms were included. Compassionate care, compassion, and empathy in health care delivery, respectful health care, and caring behavior exhibited by health professionals as well as related concepts were explored in this review.
C: context	All countries in the world

### Study Selection Criteria

Textbox 1 outlines the inclusion and exclusion criteria for the scoping review.

**Inclusion and exclusion criteria for the scoping review.**

**Inclusion criteria**

- Focus on health care providers or health professionals
- Report on health care practices or any health care services provided to any community
- Published from 2014 to September 2020
- Qualitative and quantitative studies

**Exclusion criteria**

- Publication in a non-English language
- Studies for which a full-text article could not be obtained (ie, studies with no full-text were excluded after repeatedly contacting the authors)

### Data Extraction and Management

Data were extracted using a standardized data extraction spreadsheet. The data extraction sheet included study characteristics such as author name, year, country, types of health services with CRC, the purpose of exercising CRC, study population, study design, and publication year. Data were extracted by 2 of the authors (AN and DAA) independently. The level of agreement between the 2 reviewers was measured using the Cohen kappa level of agreement [25]. The 2 authors resolved disagreements by discussion, consulting a third author (BFE) for any persistent disagreements.

### Study Selection and Reliability

Initial searches were performed by 2 review authors with extensive experience in systematic reviews. The screening of titles, abstracts, and full texts was conducted independently by 2 review authors (AN and DAA). A disagreement regarding the decision against the inclusion of articles between the 2 reviewers was resolved by consensus or the third reviewer (BFE).

A second reviewer (DAA) was blinded to the primary reviewer’s (AN) decision for checking article selection, data extraction, and risk of bias assessment stages of the reviews. Any differences of opinion were discussed; otherwise, a third reviewer (BFE) was available to arbitrate any issues that remained unresolved.

### Data Analysis

The methodological framework for the scoping review was supposed to present our narrative account of findings in 2 ways [24]; first, attention was given to the basic numerical analysis of the extent and distribution of the studies included in the review. We produced distributions for the study setting geographically, by urban or rural setting, by type of publications, and by CRC health services using tables and graphs. Second, the study findings from the existing literature are presented using thematic analysis. Then, a code book (Multimedia Appendix 1) and its definitions were prepared in a separate Word document. Our narrative literature was then structured around the themes derived from the study results. The themes that emerged from the study were (1) facilitators for CRC health care delivery, (2) barriers to CRC health care delivery, (3) disrespectful and abusive care encountered by patients, and (4) perspectives on CRC health care delivery.

## Results

### Flow of the Search and Study Characteristics

**Figure 1:**
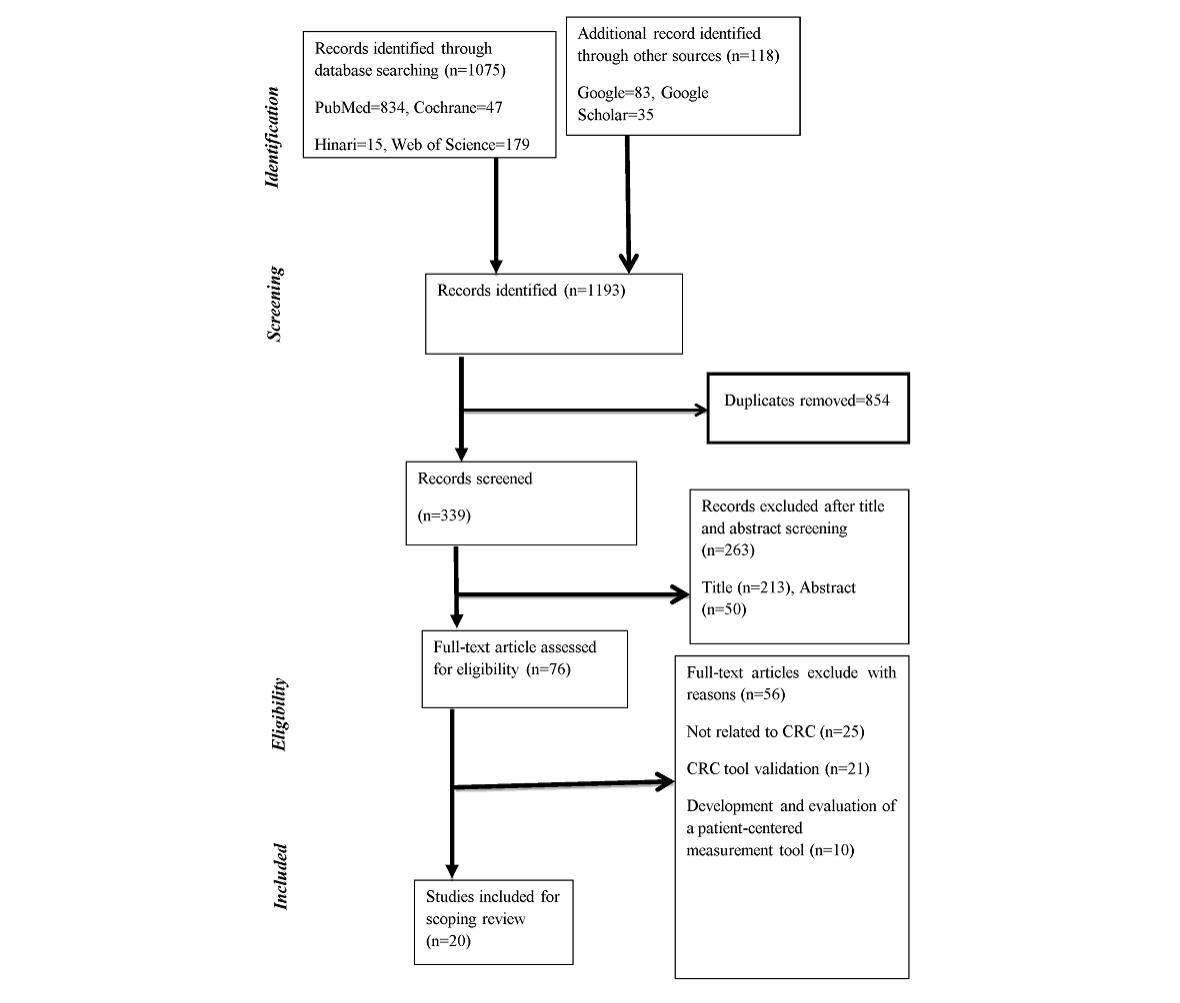
Flow diagram for the scoping review process adapted from the PRISMA (Preferred Reporting Items for Systematic reviews and Meta-Analyses) statement. CRC: compassionate, respectful, and caring.

A total of 1193 potentially relevant studies (834 from PubMed, 35 from Google Scholar [advanced], 83 from Google, 47 from CINAHL, 15 from Hinari, and 179 from Web of Science) were generated from the initial search. After duplicates were excluded, 339 studies remained. Then, we excluded most of the potentially irrelevant papers based on the review of title (n=213) and abstract (n=50). Overall, 76 studies were eligible for full-text screening. After reading the full text, 56 studies were excluded due to not being related to CRC (n=25), a focus on CRC tool validation (n=21), and a focus only on the development and evaluation of a patient-centered measurement tool (n=10); 20 articles were retained for the final review (Figure 1).

Based on the inclusion criteria, 20 studies were included in this scoping review (Multimedia Appendix 2). The idea related to compassion in health care service delivery practice was assessed and explored via a mixed (qualitatively and quantitatively) approach in 2 studies from the perspectives of health professionals and patients [26,27].

Of the studies included in this review, 3 addressed respectful care from the perspective of both health care workers and clients, in relation to respectful maternity care, disrespect and abuse during labor and delivery services, and patient-centered communication in Burkina Faso, Nigeria, and China [28-30]. The studies showed that disrespectful and abusive health care delivery is a common challenge. Caring was also addressed in 3 studies from the perspective of health care workers and clients in Ghana, Nigeria, and Reggio Emilia, Italy. A study conducted in Nigeria showed that 93.2% of the respondents had experience with at least one form of disrespectful and abusive care [31-33].

Care and respectful care were addressed in 4 studies included in this review from the perspective of midwives, midwifery students, and clients; the studies discussed any disrespectful or abusive maternal care and respectful care during labor and childbirth from their perspective [34-37]. Denial of health care delivery, overlooking of patient-centered treatment, and low socioeconomic status were the usual problems with regard to care and respectful care. In a study conducted in Ghana, about 72% of the respondents said that maltreatment was the common problem, and 77.4% said private facilities treat their clients more respectfully than public facilities [37]. Care and compassion in health care delivery were stated in 2 studies on the principles of compassion in health care practices and from the hospitalized patient viewpoint [38,39]. Only 1 study was conducted on compassionate and respectful health care delivery and showed that the magnitude of disrespect was 43% [40].

The concepts of compassion, respect, and care were assessed in 5 studies from the perspectives of health care providers, clients, and educators of health professionals [41-45]. In this review, a study in Ethiopia on the experience of CRC showed that 55% was good and the remaining 45% was poor. Patients’ perceptions toward CRC were found to be poor in 56% of the participants [42].

### Characteristics of the Included Studies

In this review, 14 studies originated from Africa, namely Ethiopia, Ghana, Nigeria, Burkina Faso, and South Africa [27-29,31,32,34-37,40-43,45], and 5 studies were from other continents, in the countries of Iran, the United Kingdom, European countries, and China [26,30,33,39,44]. A qualitative approach was used by 10 of the studies [28,29,31,33-36,41,43,44], 2 used a mixed approach [27,30], 5 were cross-sectional studies [32,37,40,42,45], 1 was a descriptive-analytical study [39], 1 was based on expert consensus [26], and 1 was an expert review [38]. Of the 20 studies, one-half (10/20, 50%) of the studies were from the perspective of health workers [26,28,29,35-37,41,43,44], 7 of the studies were from the perspectives of clients [27,30,32,33,39,40,42,45], and the other 3 studies were from both health workers’ and clients’ perspectives [31,34] (Table 2). The studies included in this review were published between 2014 [28] and 2020 [45].

Of the 20 studies, 8 were about the services delivered for maternal health components [28,32-36,40,45], 11 studies were about the services given for all clients [26,29-31,37-39,41-44], and 1 study was with oncology patients [27].

**Table 2:** Summary of studies included in the scoping review of compassionate, respectful, and caring (CRC) health care delivery (2014 to 2020).

Variable		Results, n (%)
**Perspectives**		
	Health professional	10 (50)
	Client	7 (35)
	Both	3 (15)
**Publication**		
	Original article	18 (90)
	Expert review	2 (10)

### Themes in the Studies

Based on the review of the articles, the primary focus of the included studies fitted broadly into 5 key themes, namely (1) the status of CRC implementation, (2) facilitators for CRC health care delivery, (3) barriers to CRC health care delivery, (4) disrespectful and abusive care encountered by patients, and (5) perspectives on CRC.

#### Status of CRC Implementation

Compassionate care is about empathetic conversations with the client through an evidence- based framework for the safest and most trustful interaction. This evidence-based framework takes into account the transcultural settings and needs. Health care is provided for any individual who is in need of the services in a good, compassionate manner for all ages and all illnesses, taking into account the transcultural settings and the needs, wishes, and expectations of most of the users [26]. Treating the patient as a person, not just a disease, and listening attentively to patients are the elements of compassionate care with the highest rating [27]. In this review, the status of compassionate and respectful maternity care and associated factors in health facility–based childbirth was identified, and the prevalence of experienced compassionate and respectful maternity care was 57% [40].

Patient-centered care was examined with the elements of a patient-centered care framework: respecting patients’ values, access to care, information access, patient empowerment, family and friend involvement, emotional support, and continuity of care [46]. This review showed that patients experienced at least one form of disrespect and abuse during health care service utilization [32,45].

#### Facilitators for CRC Health Care Delivery

There were a number of facilitators that enabled the health care delivery system by integrating the elements of CRC into every component of health care. Shared decision-making, fundamental principles of compassionate care, knowledge and technical skill building, in-service education, monitoring and accountability, sociodemographic characteristics of individuals, and ensuring comprehensiveness of care are some of the facilitators coded under these themes.

#### Shared Decision-making

Health care is provided for any individual who is in need of the services and in a compassionate manner for all ages and all illnesses, taking into account the transcultural settings and the needs, wishes, and expectations of most of the users. Considering shared decision-making as a cornerstone of evidence-based health care practice can be examined as moving beyond simply having empathetic conversations to developing a compassionate, evidence-based framework for safe and trusting interaction with the client [26]. “Treat you as a person, not just a disease, and listen attentively to you” were elements of compassionate care with the highest rating [27].

#### Fundamental Principles of Compassionate Care

This review found that the fundamental principles of compassionate care, such as trust, dignity, and respect, as well as effective communication skills and collaboration with patients and their families, are core requirements for the essential skills cluster “Care, Compassion, and Communication” and are required for the delivery of high-quality care [38]. All health professionals working across all health care settings should be closely aware of the concept of compassionate care.

#### Knowledge and Technical Skill Building, as Well as In-Service Education

Newly qualified health professionals must acquire sufficient knowledge and technical skills to care for patients and develop and demonstrate the attitudes and interpersonal attributes that characterize compassionate care [30].

Nurses need to see the patient’s care needs and expectations from the patient’s point of view and pay more attention to the aspects that are more important for the patients. Paying more attention to compassionate nursing care in nursing textbooks is recommended, and nurses should receive in-service education in this regard [39]. The status of compassionate and respectful maternity care and associated factors in health facility–based childbirth was identified, and the results indicated that we should do more on the issue [40].

Clients expressed moderate enthusiasm for patient-centered communication and strong preferences concerning physician respect for the patient perspective but less concern for power sharing. This means that patients were more concerned about doctors exhibiting a caring perspective than power sharing. To have respectful health care delivery services, health care providers need training on how to incorporate elements of respectful maternity care into practice, including skills for rapport building and counseling [29].

#### Monitoring, Accountability, and Sociodemographic Characteristics.

Frequent monitoring of care provision in health care facilities is needed to eliminate the incidence of disrespectful and abusive care. Midwives have described disrespectful and abusive care as the provision of inadequate care, overlooking of patient-centered care, and verbal, physical, and psychological abuse. Disrespectful and abusive care is facilitated by socioeconomic inequalities, provider perception, victim blaming, and other health system–related factors [35].

The provision of high-quality, patient-centered, respectful care for all patients, including laboring women, by health care professionals who were committed to providing a safe birthing experience for the patients and believed that yelling, shouting, and even hitting women in order to ensure a positive outcome was justified, understood, and perhaps even appreciated by women [36].

Maltreatment is a problem in the utilization of health care services. Private health facilities treated their patients more respectfully than public facilities. The majority of midwifery students throughout Ghana witnessed disrespectful care during their training. Improving monitoring, accountability, and consequences for maltreatment within facilities to improve the care that pregnant and laboring women receive is very important to having respectful care practices [37].

#### Ensuring Comprehensiveness of Care

Patient-centered care was perceived as providing quality care, making partnerships, provision of information, patient involvement, and understanding patient preference. Patient empowerment and family and friend involvement in patient care were found to be far from the existing practice and were less common in the presence of low patient health literacy levels. Patient-centered care was examined with the elements of a patient-centered care framework: respecting patients’ values, access to care, information access, patient empowerment, family and friend involvement, emotional support, and continuity of care [31].

The development of a feasible multicomponent palliative care intervention by involving clients in the decision-making process and conducting appropriate educational processes and training for health workers was significant for meeting the needs of the clients [33].

#### Barriers to CRC Health Care Delivery

In this review, we identified a number of barriers that hinder the integration of CRC health care delivery. Some of the codes comprising the subthemes include a lack of human resource development, lack of infrastructure at the health facility, poor behavior by health workers, poor client experience at the health facility, and client sociodemographic characteristics [28-31,34,35,40,41].

From the perspective of health care professionals, the most commonly cited barriers to CRC health care delivery were busy staff, absence of close follow-ups or monitoring by the leaders; knowledge and attitude gaps; absence of an information desk, complaint handling mechanism, a mechanism to obtain client feedback, discrimination-free care, friendly care, regular health education in the health facility, and capacity building; high patient flow; bed shortages; being treated by different physicians; high staff turnover; and large numbers of patients in referral centers [27,28,30,41]. From the clients’ perspectives, the most commonly cited barriers to CRC care were abuse of clients during facility health service delivery, violation of clients’ rights, occupational status of the client, previous experience with health facility utilization, residency, time, complication during service delivery, family monthly income, and intention to use a health facility [40,45].

#### Disrespect and Abusive Care Encountered by Patients

In this review, there were many forms of disrespectful and abusive care encountered by patients or clients that need to be addressed properly. The most common forms of disrespect and abuse experienced by clients were nondignified care, lack of privacy, physical abuse, neglectful care, nonconfidential care, and nonconsented care. Clients have suggested that more attention should be paid to patients’ care needs, expectations from the patient’s point of view, and the aspects that are more important for the patients [32,33,39,40,45].

The nature of disrespect and abuse in midwifery care during labor and delivery involves the denial of the preferred birth position, denial of accompaniment, denial of care, poor clinical practice, neglect, and verbal abuse from the patients’ perspectives, as well as denial of service; verbal abuse; physical abuse like hitting, pinching, and slapping; and violation of privacy from the midwives’ perspectives and women’s experiences with care from midwives during labor and delivery, including any disrespect or abuse [34].

#### Perspectives on CRC

Perspective is an art technique that changes the space or depth of CRC on health care delivery. The way of looking at CRC is not similar for everyone (ie, the health care provider and client may look at CRC in different ways). In this review, health care providers and clients were looking at CRC health care delivery in different ways, which is very important to take into account to develop an appropriate intervention.

#### Health Care Provider Perspectives on CRC

Great emphasis on and recommendations for education (CRC attitude and value more than cognitions) are needed. Designing an educational curriculum with respect to CRC for all health professionals in a higher educational program from which health professionals will graduate is the best strategy to produce health care workers who could practice his or her profession in the appropriate and ethical way [37,43]. “Caring” was taken to mean being able to converse well, up to-date, and proficient in the field of work as well as being considerate and respectful to others. Professional midwives indicated that they have seen colleagues demonstrate uncaring behavior, educators emphasized respect as caring, and student midwives, professional midwives, and educators described caring as being a competent nurse with compassion and respect for others [43].

Positive achievements in CRC health care delivery were feedback to colleagues who did not follow the training recommendations, commitment of trained health workers, improved cleanliness in the delivery room, ample explanation given to the client before providing a diagnosis, and communication with the parturient before and during the intervention [28].

Disrespect and abuse in health care and the impact on the health and well-being of the patient were perpetrated or witnessed as a violation of human rights while highlighting the patient’s expectations of care as the basis for the subjectivity of experiences [29].

A study in the Tigray region showed that the experience of patients with CRC health care practice was reported as good by 55% of respondents. In contrast, patients’ perceptions toward CRC was found to be poor, as reported by 56% of the participants [42].

Compassionate care from the perspective of staff working in health settings wishing to provide compassionate care, on its own, was insufficient to ensure this transpired; health care providers needed to work in a setting that supports them doing this, which underpins our core concept of the compassionate care flow. As “professional” compassion was associated with the intention to improve patient health and participants’ roles within health care, a compassionate care flow could be enhanced by defenders (eg, supportive colleagues, seeing the patient as a person, drawing on their faith) or depleted by drainers (ie, competing demands on time and resources), through their impact on professional compassion [44].

#### Client Perspectives on CRC

Clients who visit a health facility for different health care services stated that compassion and respectful health care services provided by health personnel encouraged them to visit again for any other medical check-up. Many of the clients wanted to be treated by one physician based on their choice, but most of the clients reported that they were treated by different physicians without being asked their choice [27].

Clients expressed strong preferences concerning physician respect for patient perspectives rather than for power sharing. Younger and highly educated patients were more likely to prefer patient-centered communication, and highly educated patients paid more attention to power sharing [30].

## Discussion

### Principal Findings

This systematic scoping review explored the available literature on CRC health care delivery, based on the perspectives of health professionals and patients or clients in the global context. In this review, as a country, Ethiopia was the most represented, which might be linked with the fact that Ethiopia initiated the design of a 5-year Compassionate, Respectful and Caring Health Services Implementation Strategy as one of the national top priorities set under the health sector transformation plan [12]. In this review, 90% (18/20) of all articles were published in the last 5 years, suggesting that patients or clients and health professionals increasingly view patient-centered communication, compassion, respect, and caring as essential for good health service utilization [26,29-31,33,39,43-45].

In addition, the current review delivers some insight into the status of compassion, suggesting that compassionate care is about empathetic conversations with the client through an evidence-based framework for the safest and trustful interaction and treating patients as a person, not just a disease, and listening attentively were elements of compassionate care with the highest rating [26,27].

CRC health care delivery behaviors have direct impacts on health-seeking behaviors and overall health outcomes [47]. Compassion does not depend on pre-existing relationships; rather, it is delivered through a long-term relationship with individuals. Compassion consists of specific skills and actions aimed at the enhancement of multifactorial suffering, namely, acknowledging, responding to, understanding, and actively addressing the suffering of another [47].

This review on the concepts of respectful health care delivery showed that patients were more concerned about doctors exhibiting caring perspectives than power-sharing. There was more emphasis on patient-centered communication and strong preferences concerning physician respect for patient perspectives and less concern for power-sharing [30]. High staff turnover and large numbers of patients in referral centers were the main challenges observed for respectful health care delivery [29].

Disrespectful and abusive practices were witnessed as a violation of human rights while highlighting women’s expectations of care as the basis for the subjectivity of experiences [29]. Respectful health care is the factor most neglected in health care provision [48] but that increases client satisfaction and affects the health-seeking behaviors of the community. A practice of respectful health care is a set of safe health facilities for health care services where the clients or service users are valued, recognized, treated fairly, have clear expectations, and appropriately access services, as needed. When there is a practice of respectful health care delivery at the health institution, the likelihood of the community using the facility could be increased in a remarkable way [49].

A patient-centered framework is an essential element for the patient, one that is guided by respect for the patient’s values, access to care, access to information, patient empowerment, involvement of family and friends, emotional support, and continuity of care [12]. Engagement of clients influences both the overall health care services and improves health care provision because both clients and health care providers feel respected, listened to, and empowered [50,51].

CRC health care delivery is essential for the successful utilization of health services. The findings from this review suggest that CRC health care delivery might have a positive effect on specific health care service utilization, including increased service satisfaction and sustainability of service utilization. The barriers toward implementation of CRC health care practice in this review from the perspective of health care professionals are busy staff; absence of close follow-ups or monitoring by the leaders; knowledge and attitude gaps; and absence of an information desk, a complaint handling mechanism, mechanism for obtaining client feedback, and regular health education in a health facility [41]. This is in agreement with a review conducted in a clinical health care setting [52].

In this review, in a study on caring from the perspectives of undergraduate student midwives, professional midwives, and teachers of midwifery, participants described caring as being a competent nurse with compassion and respect for others [43]. This finding could help to design an integrated curriculum for health professional teaching and to have a strong CRC norm in health care delivery. Similarly, a cross-sectional study conducted in Ethiopia on the status of CRC with perspective clients showed that the experience and perception of patients toward CRC health care practice were good, which means that clients had experience with CRC health care service from a health care provider [42]. On the other hand, there are some barriers for the implementation of CRC in health care practices such as busy staff due to overloaded work; absence of close follow-ups or monitoring by the leaders; knowledge and attitude gaps; and absence of an information desk, a complaint handling mechanism, mechanism for obtaining client feedback, regular health education in a health facility, and capacity building. Therefore, we recommend that enhancing CRC health care delivery in the health care system requires empirical teaching methods as a baseline that engage the learner professionally, ethically, and personally, because compassion, respect, and caring are rooted in the nature of the students and the actualization of these qualities within health care service delivery practices, in addition to considering periodical refresher and follow-up training for those who are employed in the health care delivery system.

### Strengths and Limitations

Scoping reviews are broad in nature and provide an overview of existing literature regardless of quality, providing a broader and more contextual overview than systematic reviews. Use of the PRISMA-ScR was a strength of this scoping review. A formal assessment of methodological quality is not undertaken when conducting a scoping review and synthesis of the incorporated studies. Including papers published only in English was another limitation of this review.

### Conclusion

This scoping review showed the status of disrespectful and abusive care encountered by the patients, facilitators of and barriers to CRC health care delivery behaviors, and different perspectives. The status of CRC health care delivery remains challenging and needs strong involvement from different organizations from all disciplines. Pre-service education with full CRC competencies (ie, higher education institutes) should include CRC as one component of the curriculum for health professionals, as well as frequent in-service training and inclusion of CRC elements in human resource selection, performance management, and incentive systems, including career advancement, deployment opportunities, and labor division at health facilities.

## Appendix

Multimedia Appendix 1 Codebook for thematization of the study.

Multimedia Appendix 2 Study characteristics.

## Abbreviations

CRC: compassionate, respectful, and caring
JBI: Joanna Briggs Institute
PCC: participant, concept, and context
PRISMA-ScR: Preferred Reporting Items for Systematic reviews and Meta-Analyses extension for Scoping Reviews
UHC: universal health coverage
WHO: World Health Organization
