# Supplementary material for: Status of Compassionate, Respectful, and Caring Health Service Delivery: Scoping Review
Source: JMIR Hum Factors. 2022 Feb 7;9(1):e30804. doi: 10.2196/30804 (PMC8863364; doi:10.2196/30804)
Supplement: Multimedia Appendix 1 [file humanfactors_v9i1e30804_app1.docx]

**Multimedia Appendix 1: Themes and sub themes with its definitions**

| **Themes** | **Sub-themes** | **Definitions** | **References** |
| --- | --- | --- | --- |
| **Facilitators for CRC health care delivery** | Shared-decision making | Safe and trusting interaction with the client | Ref.19 and 20 |
|  | Fundamental principles of compassionate care | trust, dignity, respect, effective communication skills and collaboration with patients and their families | Ref.31 |
|  | knowledge and technical skill buildings | develop and demonstrate the attitudes and interpersonal attributes, training on how to incorporate elements of respectful care into practice including skills for rapport building and counseling | Ref.23 |
|  | In-service education | Paying more attention to compassionate care in textbooks | Ref.22,32 and 33 |
|  | Socio-demographic | Socio-economic inequalities, provider perception and victim blaming, and health system related factors | Ref.29 |
|  | Monitoring and accountability | Improve monitoring, accountability, and consequences for maltreatment within facilities | Ref.28 and 30 |
|  | Ensuring comprehensiveness of care | Patient centered care (respect patient’s values, access to care, information access, patient empowerment, family and friend’s involvement, emotional support and continuity of care), Patient empowerment and family and friend involvement in patient care. | Ref.24 |
| **Common** **Barriers in CRC health** **care delivery** | Lack of human resource development | busy staff, absence of close follows up /monitor by the leaders, knowledge, attitude gap, absence of information desk, complaint handling mechanism, and clients’ feedback obtaining mechanisms, regular health education in health facility, lack of capacity building, being treated by different physicians , and high staff turnover . | Ref,21,22,24,25 |
|  | Lack of infrastructure at health facility | high patients’ flow, lack of toilet, lack of water, lack of room for privacy | Ref.21,22,24,35 |
|  | Bad behavior of health workers | abuse of clients during facility health service delivery, violation of clients’ rights | Ref.34,39 |
|  | Bad experience of clients at health facility | previous experience of health facility utilization, complication during service delivery, and intention to use health facility | Ref.34,39 |
|  | Clients socio-demographic condition | occupational status of the client, residency, time, family monthly income | Ref.34,39 |
| **Disrespectful and abusive care encountered by patient** | Disrespect care | non-dignified care, neglectful care, providing friendly care | Ref.26,27,33,34,39 |
|  | Abusive care | lack of privacy, physical abuse, non-confidential care, non-consented care, discrimination free care | Ref.26,27,33,34,39 |
| **Perspectives of CRC** | Health care provider perspectives of CRC | The study participant was health care provider and they reflect their feeling regarding the CRC health care delivery | Ref.30,31,37,22,23,36,38 |
|  | Client perspectives of CRC | The study participant was clients/service users and they reflect their feeling regarding the CRC health care delivery | Ref.20 and 23 |
| **status of CRC implementation** | Compassion | Taking into account the transcultural settings and the needs. The health care provided for any individual who is in need of the services in a good compassion manner of care for all ages and all illnesses by taking into account the transcultural settings and the needs, wishes and the expectation of most of the users; | Ref.19 and 20 |
|  | Respectful | Respect patient’s values | Ref.39 |
|  | Caring | Access to care, information access, patient empowerment, family and friend’s involvement, emotional support and continuity of care. | Ref.39 |
|  | Compassion, Respectful and Caring | Empathetic conversations with the client through evidence based frame for good interaction, respect values and accessing every need of care together. | Ref.19,20,25,38 and 39 |
