# Supplementary material for: Status of Compassionate, Respectful, and Caring Health Service Delivery: Scoping Review
Source: JMIR Hum Factors. 2022 Feb 7;9(1):e30804. doi: 10.2196/30804 (PMC8863364; doi:10.2196/30804)
Supplement: Multimedia Appendix 2 [file humanfactors_v9i1e30804_app2.docx]

| **Multimedia Appendix 2: Study characteristics** | | | | | | | | | |
| --- | --- | --- | --- | --- | --- | --- | --- | --- | --- |
| **S.N** | **Author.** | **year** | **country** | **perspective** | **Population for whom the service delivered** | **Sample size** | **design** | **Aim/purpose** | **Key finding** |
| 1 | Wassihun B. | 2108 | Ethiopia | Patient | Mothers who gave birth | 284 | Cross sectional | Assessing the status of compassionate and respectful maternity care and associated factors in health facility-based childbirth | - The prevalence of disrespect and abuse during childbirth was 43% among the women studied. Abuse of mothers during facility child birth is a health facility failure and a violation of women’s rights as well as an important barrier to women seeking institutional delivery |
| 2 | Berhe H. | 2018 | Ethiopia | All Health professionals | All | 40 | qualitative | Exploring Barriers Towards Implementation of Caring, Respectful and Compassionate Healthcare Practice | - Busy staff, absence of close follows up /monitor by the leaders, knowledge .attitude gap, absence of information desk, complaint handling mechanism, clients’ feedback obtaining mechanisms, regular health education in health facility. Lack of capacity building |
| 3 | Berhe H. | 2017 | Ethiopia | Patient | All | 1386 | Cross sectional | assessing Status of Caring, Respectful and Compassionate Health Care Practice in at health facilities | - Experience of patients towards caring, respectful and compassionate health care practice was found to be good in 55% of respondents and poor in the rest 45% respondents. Similarly, patients’ perception towards CRC was assessed and found to be good and poor in 56% of the participants |
| 4 | Ouédraogo A. | 2014 | Burikinafaso | health professional | mothers during labour and delivery | 46 | qualitative (observed) | assessing **Respectful** maternity care in health facilities : The experience of the Society of Gynaecologists and Obstetricians | - The SOGOB pilot program to improve quality of maternity care by combining good practices with respectful behaviours merits expansion to health facilities across the country - The main challenges observed included high staff turnover and large numbers of patients in referral centres. |
| 5 | Burrowes S. | 2017 | Ethiopia | both midwifes and patient | mothers during labour and delivery | 45 | qualitative | examine the nature of disrespect and abuse in midwifery care during labor and delivery in the study setting and Examine women’s experiences of care from midwives during labor and delivery, including any disrespect or abuse | - Denial of preferred birth position , Denial of accompaniment ,denial of care , Poor clinical practice neglected, and verbal abuse on patients perspective whereas denial of service, verbal abuse ,physical abuse like hitting ,pinching, slapping and violation of privacy from midwives perspective |
| 6 | Dzomeku VM | 2020 | Ghana | Midwives perspective | Mothers | 15 | qualitative | Exploring the experiences and views of midwives on disrespectful and abusive maternal care in their professional practice in Ghana | - Frequent in-service training on respectful maternity care and monitoring of care provision in healthcare facilities are needed to eliminate the incidence of disrespect and abuse care - The midwives described D&AC as the provision of inadequate care and the overlooking of patient-centred care, and verbal, physical, and psychological abuse. - socio-economic inequalities, provider perception and victim blaming, and health system related factors facilitate D&AC |
| 7 | Rominski S | 2016 | Ghana | Midwives perspective | mothers during labour and delivery | 83 | Qualitative | Examining disrespectful and abusive treatment towards labouring women from the perspective of midwifery students who were within months of graduation | - Although midwifery students highlight the importance of providing high-quality, patient-centered respectful care, they also report many forms of disrespect and abuse during childbirth. - The midwife students are dedicated to providing a safe birthing experience for their patients and felt that yelling, shouting, and even hitting women in order to ensure a positive outcome was justified and understood, and maybe even appreciated, by women |
| 8 | Jacqueline Bloomfield | 2015 |  |  | All |  |  | To explore fundamental principles of compassionate care and provides examples of how such principles may be incorporated into nursing care across a range of healthcare settings | - All nurses working across all healthcare settings should Closely entwined with the concept of compassionate care with the elements of trust, dignity, respect, effective communication skills and collaboration with patients and their families are core requirements form the basis of the essential skills cluster Care, Compassion and Communication, and are essential for the provision of high quality care. - Newly qualified nurses must acquire sufficient knowledge and technical skills to care for patients, and develop and demonstrate the attitudes and interpersonal attributes that characterise compassionate care |
| 9 | Enkeleint-Aggelos Mechili | 2018 | European | Primary Health care professionals | All | 69 | Expert Consensus Meeting | To provide good and affordable, comprehensive, person-centred, integrated and compassionate care for all ages and all ailments, taking into account the transcultural settings and the needs, wishes and expectations of the newly arriving refugees. | - Considering shared-decision making as a cornerstone of evidence based practice, it can be examined as moving further beyond simply having empathetic conversations to developing a compassionate, evidence-based frame for safe and trusting interaction. |
| 10 | Asghar Dalvandi | 2017 | Iran | patients | All | 300 | Descriptive analytical method | To determine the importance and extent of providing compassionate nursing care  from the hospitalised patients’ viewpoint in educational hospitals in Kermanshah-Iran 2017 | - Nurses need to see the patient’s caring needs and expectations from the patient’s point of view and pay more attention to the aspects that are more important for the patients. - Paying more attention to compassionate nursing care in nursing textbooks is recommended, and the nurses should receive in-service educations in this regard |
| 11 | Tamiru Bogale | 2017 | Ethiopia | Health care provider,admin.staff and patients | All | 32 | Qualitative case study | To examine the scope of patient centered care practice and to identify potential factors that drive or hinder patient centered care practice in public hospitals of Benishangul Gumuze regional state, South West Ethiopia | - Patient-centered care was perceived as; providing quality care, making partnership, provision of information, patient involvement and understanding patient preference. - Patient empowerment and family and friend involvement in patient care were found far from the existed practice and which was favoured by low patient health literacy levels. |
| 12 | Cheryl A. Moyer | 2014 | Ghana | midwifery students throughout Ghana | All | 929 final-year students | cross-sectional survey | To determine what midwifery students throughout Ghana were witnessing, perceiving, and  learning with regard to respectful care during labour and childbirth | - 72.0% said maltreatment was a problem in Ghana and 77.4% said women are treated more respectfully in private than public facilities. - Majority of midwifery students throughout Ghana witness disrespectful care during their training. - Improve monitoring, accountability, and consequences for maltreatment within facilities to improve the care that pregnant and labouring women receive |
| 13 | Ogunlaja AO | 2017 | Nigeria | Patien/client | Pregnant women | 438 pregnant women | cross-sectional study | To determine whether they had experienced disrespect and abuse during  maternity care in the past or not | - 93.2% (408) of the respondents had experienced one form of disrespect and abuse. - There was a significant statistical relationship between occupation, educational status, parity and experience of disrespect and abuse in maternity care amongst the respondents - The most common forms of disrespect and abuse experienced by these women were non-consented care and non-confidential care |
| 14 | Merkeb Zeray | 2018 | Ethiopia | Patients | Oncology patients | 423 oncology patiens | Mixed quan.With qual. | To measure compassionate care practice among oncology  patients at Tikur Anbessa Specialized Hospital. | - The overall compassionate care practice was found to be 45.7% and high patients’ flow, bed shortage and being treated by different physicians were among the factors that identified to be a barrier for compassionate care practicing |
| 15 | Joy Orpin | 2018 | Nigeria | Health care provider | All | 16 | Qualitative approach | To explore healthcare providers’ perspectives of disrespect and abuse in maternity care and the impact on  women’s health and well-being | - Health care providers considered disrespectful and abusive practices perpetrated or witnessed as violation of human rights, while highlighting women’s expectations of care as the basis for subjectivity of experiences. - Healthcare providers need training on how to incorporate elements of respectful maternity care into practice including skills for rapport building and counselling |
| 16 | S Tanzi | 2020 | Reggio Emilia, tally | Health care provider | mothers during labour and delivery | 31 | single holistic case study design | To describe a palliative care unit’s consultation and assistance intervention at the request of an  Infectious Diseases Unit during the COVID-19 pandemic, determining what changes needed to be made in delivering palliative care. | - Our Palliative Care Unit developed a feasible 18-day multicomponent consultation intervention. Three macro themes were identified: (1) new answers to new needs, (2) symptom relief and decision-making process, and (3) educational and training issues. |
| 17 | Mmajapi E.T. Masala-Chokwe | 2015 | South Africa | undergraduate student midwives, professional midwives, and the educators teaching | All | 38 | qualitative and exploratory | Exploring the meaning of caring from the perspectives of the undergraduate student midwives, the professional midwives, and the educators teaching midwifery in Tshwane | - ‘Caring’ was taken to mean being well conversant, up to-date and proficient in the field of work as well as considerate and respectful to others. The professional midwives indicated that they have seen colleagues demonstrate uncaring behaviour whilst educators emphasised respect as caring - The student midwives, professional midwives and educators described caring as being a competent nurse with compassion and respect for others |
| 18 | Ting, X. | 2016 | China | patient | All | 317 for quan. and 20 for qual. | Quantitative and qualitative | To investigate patient perceptions of patient-centered communication (PCC) in doctor-patient consultations and explore barriers to PCC implementation in China | - Patients expressed moderate enthusiasm for PCC in China. They expressed strong preferences concerning physician respect for patient perspective, but less concern for power sharing. - Patients were more concerned about doctors exhibiting caring perspective than power sharing - Younger and highly educated patients were more likely to prefer PCC and highly educated patients paid more attention to power sharing. |
| 19 | Stephanie Tierney | 2015 | United kingdom | Health professionals | All | 36 | qualitative | - To explore compassionate care from the perspective of staff working in health settings. - Explored their understanding of compassionate care and experiences of it in practice. | - Wishing to provide compassionate care, on its own, was insufficient to ensure this transpired; HCPs needed to work in a setting that supported them to do this, which underpins our core concept – the compassionate care flow. - As ‘professional’ compassion, was associated with the intention to improve patient health and participants’ role within healthcare. The compassionate care flow could be enhanced by defenders (e.g. supportive colleagues, seeing the patient as a person, drawing on their faith) or depleted by drainers (i.e. competing demands on time and resources), through their impact on professional compassion. |
| 20 | Bekele | 2020 | Ethiopia | Client | Mothers with under 1 year child | 321women | Cross sectional | To measure the magnitude of disrespect and abusive behaviours of health professionals during child birth and associated factors | - The most common forms of disrespect and abuse encountered by the mothers were: unconsented care, non-dignified care, lack of privacy, physical abuse, and neglectful care. - Mothers occupational status, increasing number of ANC visit and giving birth in a hospital setting were significantly associated with disrespect and abuse during facility based child birth. |
